# Visit-to-visit variability of fasting plasma glucose as predictor of ischemic stroke: competing risk analysis in a national cohort of Taiwan Diabetes Study

**DOI:** 10.1186/s12916-014-0165-7

**Published:** 2014-09-26

**Authors:** Cheng-Chieh Lin, Chun-Pai Yang, Chia-Ing Li, Chiu-Shong Liu, Ching-Chu Chen, Wen-Yuan Lin, Kai-Lin Hwang, Sing-Yu Yang, Tsai-Chung Li

**Affiliations:** Department of Family Medicine, China Medical University Hospital, Taichung, Taiwan; Department of Medical Research, China Medical University Hospital, Taichung, Taiwan; School of Medicine, College of Medicine, China Medical University, Taichung, Taiwan; Department of Neurology, Kuang Tien General Hospital, Taichung, Taiwan; Department of Nutrition, Huang-Kuang University, Taichung, Taiwan; Graduate Institute of Integrated Medicine, College of Chinese Medicine, China Medical University, Taichung, Taiwan; Division of Endocrinology and Metabolism, Department of Medicine, China Medical University Hospital, Taichung, Taiwan; School of Chinese Medicine, College of Chinese Medicine, China Medical University, Taichung, Taiwan; Department of Public Health, Chung Shan Medical University, Taichung, Taiwan; Graduate Institute of Biostatistics, College of Management, China Medical University, Taichung, Taiwan; Department of Healthcare Administration, College of Medical and Health Science, Asia University, Taichung, Taiwan

**Keywords:** Glucose variation, Ischemic stroke, Type 2 diabetes

## Abstract

**Background:**

Glycemic variation as an independent predictor of ischemic stroke in type 2 diabetic patients remains unclear. This study examined visit-to-visit variations in fasting plasma glucose (FPG), as represented by the coefficient of variation (CV), for predicting ischemic stroke independently, regardless of glycated hemoglobin (HbA_1_c) and other conventional risk factors in such patients.

**Methods:**

Type 2 diabetic patients enrolled in the National Diabetes Care Management Program, ≥30 years old and free of ischemic stroke (n = 28,354) in 2002 to 2004 were included, and related factors were analyzed with extended Cox proportional hazards regression models of competing risk data on stroke incidence.

**Results:**

After an average 7.5 years of follow-up, there were 2,250 incident cases of ischemic stroke, giving a crude incidence rate of 10.56/1,000 person-years (11.64 for men, 9.63 for women). After multivariate adjustment, hazard ratios for the second, third and fourth versus first FPG-CV quartile were 1.11 (0.98, 1.25), 1.22 (1.08, 1.38) and 1.27 (1.12, 1.43), respectively, without considering HbA_1_c, and 1.09 (0.96, 1.23), 1.16 (1.03, 1.31) and 1.17 (1.03, 1.32), respectively, after considering HbA_1_c.

**Conclusions:**

Besides HbA_1_c, FPG-CV was a potent predictor of ischemic stroke in type 2 diabetic patients, suggesting that different therapeutic strategies now in use be rated for their potential to (1) minimize glucose fluctuations and (2) reduce HbA_1_c level in type 2 diabetic patients to prevent ischemic stroke.

## Background

Diabetes ranks as a leading cause of incident ischemic stroke [1-3]. Epidemiological study confirms that diabetes independently raises the risk of ischemic stroke, the relative risk ranging from 1.8- to nearly 6-fold [3]. A glycated hemoglobin (HbA_1_c) level of <7.0% is recommended by the American Diabetes Association (ADA) to prevent microvascular complications in type 2 diabetes patients [4,5]. Whether such control reduces risk of cardiovascular events and stroke remains unclear [5]. This has prompted large-scale studies to gauge macrovascular complications (including ischemic stroke) in such cases. Recent attention has targeted oscillating glucose levels possibly superimposed on HbA_1_c in affecting risks of complications [6-8]. Both *in vitro* and animal studies confirm that oscillating plasma glucose has a greater effect on endothelial function and oxidative-stress generation than constant high glucose [9,10]. Human studies correlate coefficient of variation (CV) of fasting plasma glucose (FPG) to outcome in type 2 diabetic patients, focusing on all-cause or cause-specific mortality [11-15]. Glycemic variation, determined by FPG-CV, as an independent predictor of ischemic stroke in type 2 diabetic patients remains unclear. Our study examined whether FPG variation, as measured by CV, showed a significant independent clinical association with ischemic stroke, regardless of HbA_1_c and other conventional risk factors.

## Methods

### Study population

**Figure 1:**
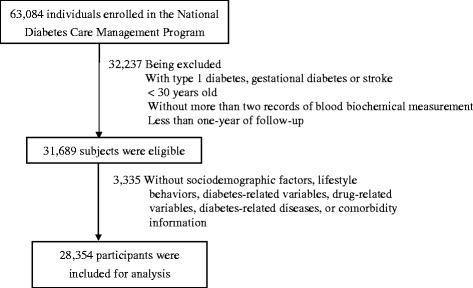
**Flowchart of recruitment procedures for the current study.**

This retrospective cohort, encompassing all enrollees in a National Diabetes Care Management Program (NDCMP) in Taiwan, is a population-based study of 63,084 ethnic Chinese type 2 diabetic patients enrolled in the NDCMP in Taiwan during 2002 to 2004. Date of entry into NDCMP was defined as the index date. NDCMP is a case management program established by the National Health Insurance (NHI) Bureau in 2002. Those with clinically diagnosed diabetes based on ADA criteria (International Classification of Disease, 9th Revision, Clinical Modification (ICD-9- CM) diagnosis code 250) were recruited without restriction for anti-diabetes medication. Type 2 diabetic patients treated with diverse insulin sensitizers, insulin secretagogues and insulin regimens were included. For diagnosis of a new patient, an individual with FPG >126 mg/dl (or 7.0 mmol/L) or plasma glucose ≥200 mg/dl (or 11.1 mmol/L) during an oral glucose tolerance test (OGTT) repeats the test on a different day to increase the validity of the diabetes diagnosis. Exclusion criteria were type 1 diabetes (ICD-9-CM code 250.x1/x3), gestational diabetes (ICD-9-CM code 648.83) and stroke (ICD-9-CM code 430–438), as well as being younger than 30 years of age. We enrolled patients with more than two recorded follow-ups of at least one year to rate FPG variability; 31,689 were eligible. Excluding those with missing data left 28,354 for analysis (Figure 1). We compared baseline characteristics between the patients included and those excluded using standardized mean differences. All standardized mean differences were less than 0.1 standard deviations (SD), indicating a negligible difference in means or proportions between groups. The study was approved by the China Medical University Hospital Ethical Review Board.

### Data sources for baseline and follow-up assessments

As of March 1995, Taiwan’s government launched an NHI program that covered approximately 99% of its population of 23.74 million in 1999 [16]. By the end of 2010, NHI contracted with 100% of hospitals and 92% of clinics island-wide [17]. The database includes patient demographics, diagnoses, and prescriptions in hospital and outpatient claims; claims data are randomly audited by the NHI Bureau. Expert reviews on a random sample for every 50 to 100 ambulatory and inpatient claims in each hospital and clinic are conducted quarterly to enhance the validity of claim data, and a severe penalty is imposed for a false diagnostic report by the NHI Bureau. This study used datasets for inpatient care by admission and outpatient visits during 2002 to 2010. Individuals in Taiwan carry unique personal identification numbers (PIN). For security and privacy purposes, patient identity data is scrambled cryptographically by the National Health Insurance Research Database (NHIRD). All NHI datasets can be interlinked with the PIN of each individual. Data comprise information for all insured subjects with regard to demographic data, date and source of diagnosis, ambulatory care, inpatient admission and outpatient/inpatient treatment. ICD-9-CM codes were used to identify individual health status. Due to comprehensive coverage of the NHI program, the proportion of enrollees withdrawing from NHI is very low; thus, bias due to being lost to follow-up is negligible. Enrollees underwent comprehensive assessment of their disease status and complications as well as a series of blood tests, urine tests and body measurements upon entering NDCMP. They completed standardized, computerized questionnaires administered by a case management nurse to record the previous or current status of their disease, medications and lifestyles. After a twelve-hour overnight fast, blood was drawn from an antecubital vein and sent for analysis within four hours post-collection. All patients were followed up regularly every three to six months.

### Outcome ascertainment

The primary outcome measure, determined by inpatient/outpatient claims, was based on the major ICD-9-CM discharge diagnosis code, ischemic stroke (ICD-9-CM codes 433–434). Accuracy of ischemic stroke diagnoses in NHIRD had been validated in an earlier study [18]. Accuracy of ischemic stroke diagnosis in our inpatient/outpatient claim dataset was 94%, indicating NHIRD as a valid resource for population research identifying ischemic stroke. We searched NHIRD data for ischemic stroke with at least one inpatient or three outpatient claims one year after the index date, excluding incident cases within one year, to rule out cause-and-effect. Linking the unique PIN with computerized files identified 2,250 cases, followed from one year after the index date until ischemic stroke, death or withdrawal from NHI. Other chronic conditions were tabulated for 12 months prior to enrollment, using outpatient and inpatient claims data: coronary artery disease (ICD-9-CM codes 410 to 413, 414.01 to 414.05, 414.8 and 414.9), congestive heart failure (ICD-9-CM codes 428, 398.91 and 402.x1), cancer (ICD-9-CM codes 140 to 149, 150 to 159, 160 to 165, 170 to 175, 179 to 189, 190 to 199, 200, 202, 203, 210 to 213, 215 to 229, 235 to 239, 654.1, 654.10, 654.11, 654.12, 654.13 and 654.14), atrial fibrillation (ICD-9-CM code 427.31), hyperlipidemia (ICD-9-CM code 272), hypertension (ICD-9-CM codes 401 to 405), chronic hepatitis (ICD-9-CM codes 571, 572.2, 572.3, 572.8, 573.1, 573.2, 573.3, 573.8 and 573.9), chronic obstructive pulmonary disease (ICD-9-CM codes 490 to 496) and hypoglycemia (ICD-9-CM code 251).

### Statistical analysis

The CV of FPG measurements from outpatient visits within the first year of the index date for each patient was calculated. FPG-CV was calculated only when more than two FPG measurements were performed in the first year. To adjust for the possibility that the number of visits may affect variation, the CV value was divided by the square root of the ratio of total visits divided by total visits minus 1 [19]. Patients were grouped into quartiles according to FPG-CV. To rule out a possible specific threshold of FPG-CV impacting the significance of the findings, sensitivity analysis classified patients into 10 subgroups according to deciles of FPG-CV. Kaplan-Meier cumulative incidence plots were derived. To weigh competing risk of death, an extended Cox proportional hazards model of competing risk data on stroke incidence fitted a proportional subdistribution hazards regression model with weights for subjects who underwent competing risk event of death, according to an extension of the Lunn and McNeil method [20]. Extended Cox proportional hazard models with competing risks served to evaluate the association between FPG-CV categories and incident ischemic stroke. Hazard ratios (HRs) and 95% confidence intervals (CI) adjusted for age, gender and multiple variables. Multivariate models (1) adjusted for age (continuous) and gender; (2) additionally adjusted for tobacco (yes/no), alcohol (yes/no), duration of diabetes, type of hypoglycemic drug, antihypertensive treatment (yes/no) and obesity (body mass index ≥27 kg/m^2^); and (3) additionally adjusted for coronary artery disease, congestive heart failure, cancer, hyperlipidemia, hypertension, atrial fibrillation, chronic hepatitis, chronic obstructive pulmonary disease and estimated glomerular filtration rate (eGFR). Interaction of FPG-CV and HbA_1_c was probed by adding their product terms into the full model using the likelihood ratio test for significance (set at two-tailed *P* ≤0.05). All analyses were performed with the SAS statistical package for Windows (Version 9.3, SAS; Cary, NC, USA).

## Results

**Figure 2:**
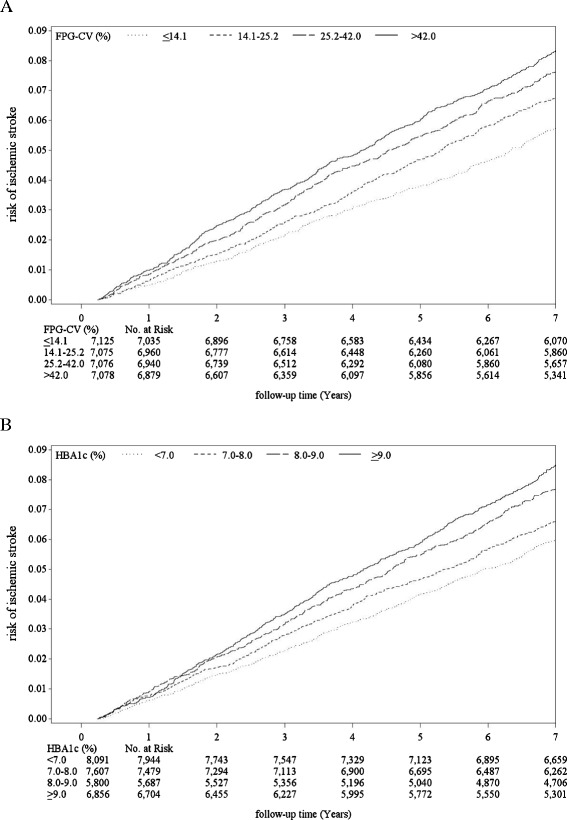
**Risks of ischemic stroke. (A)** FPG-CV and **(B)** HbA_1_c. Log-rank test, all *p* <0.001. CV: coefficient of variation; FPG: fasting plasma: glucose; HbA_1_c, glycated hemoglobin.

After an average 7.5 years of follow-up, there were 2,250 incident cases of ischemic stroke in type 2 diabetic patients, giving a crude incidence rate of 10.56/1,000 person-years (11.64 for men, 9.63 for women); 5,031 died, a mortality rate of 23.61/1,000 person-years (28.24 men, 19.63 women). Table 1 shows baseline sociodemographic and clinical factors in subjects grouped according to quartiles of FPG-CV and HbA_1_c levels (<7% versus ≥7%). Lower FPG-CV was associated with higher mean age, lower mean duration of diabetes, triglycerides, fasting plasma glucose and HbA_1_c, along with lower prevalence of female gender, tobacco, alcohol, three oral hypoglycemic drugs, insulin injections, insulin injections plus oral hypoglycemic drugs, congestive heart failure, cancer, hypertension and chronic obstructive pulmonary disease, and more frequent use of one or two oral hypoglycemic drugs, hypertension drug treatment, obesity and hyperlipidemia. Those with HbA_1_c <7% had a higher mean age, lower mean duration of diabetes, triglycerides, low-density lipoprotein, and fasting plasma glucose, lower prevalence of female gender, tobacco, three or more oral hypoglycemic drugs, insulin and insulin plus oral hypoglycemic drugs, and higher prevalence of no medication, one or two oral hypoglycemic drugs, hypertension drug treatment and hypertension. The Pearson correlation coefficient between baseline FPG-CV and HbA_1_c was weak (*r* = 0.225). Figure 1 presents the Kaplan-Meier cumulative risk for ischemic stroke within subgroups defined by FPG-CV and HbA_1_c. Patients with FPG-CV >14.1% faced a higher risk (log-rank test *P* <0.001, Figure 2A), as did those with HbA_1_c ≥7.0% (log-rank test *P* <0.001, Figure 2B).

**Table 1:** **Baseline factors according to quartiles of the coefficient of variation of FPG and HbA1c levels (n = 28,354)**

	**FPG-CV (%)**					**HbA**_**1**_ **c (%)**		
**Variables**	**≤14.1**	**14.1 to 25.2**	**25.2 to 42.0**	**>42.0**	***P*** **-value**	**<7.0**	**≥7.0**	***P*** **-value**
	**(Number = 7,125)**	**(Number = 7,025)**	**(Number = 7,076)**	**(Number = 7,078)**		**(Number = 8,091)**	**(Number = 20,263)**	
*Sociodemographic factors*								
Male, number (%)	3,405 (47.79)	3,297 (46.60)	3,232 (45.68)	3,460 (48.88)	<0.001	4,232 (52.31)	9,162 (45.22)	<0.001
Age (years), mean (SD)	60.68 (11.19)	60.79 (11.10)	60.47 (11.00)	59.92 (11.41)		61.96 (11.53)	59.87 (10.98)	<0.001
*Lifestyles*, number (%)								
Tobacco	1,006 (14.12)	1,076 (15.21)	1,094 (15.46)	1,322 (18.68)	<0.001	1,186 (14.66)	3,312 (16.35)	<0.001
Alcohol	613 (8.60)	652 (9.22)	585 (8.27)	708 (10.00)	0.002	777 (9.60)	1,781 (8.79)	0.03
*Diabetes-related variables*								
Duration of diabetes (years), mean (SD)	5.89 (7.52)	6.36 (6.85)	6.90 (7.17)	6.80 (7.21)	<0.001	5.25 (6.85)	6.98 (7.28)	<0.001
Type of hypoglycemic drug use, number (%)					<0.001			<0.001
No medication	127 (1.78)	72 (1.02)	61 (0.86)	56 (0.79)		202 (2.50)	114 (0.56)	
One oral hypoglycemic drug	1,868 (26.22)	1,350 (19.08)	980 (13.85)	820 (11.59)		2,692 (33.27)	2,326 (11.48)	
Two oral hypoglycemic drugs	3,117 (43.75)	3,213 (45.41)	3,031 (42.83)	2,673 (37.76)		3,768 (46.57)	8,266 (40.79)	
Three oral hypoglycemic drugs	1,147 (16.10)	1,332 (18.83)	1,451 (20.51)	1,321 (18.66)		873 (10.79)	4,378 (21.61)	
>3 oral hypoglycemic drugs	290 (4.07)	364 (5.14)	409 (5.78)	416 (5.88)		144 (1.79)	1,335 (6.59)	
Insulin	93 (1.31)	102 (1.44)	202 (2.85)	335 (4.73)		145 (1.79)	587 (2.90)	
Insulin + oral hypoglycemic drug	483 (6.78)	642 (9.07)	942 (13.31)	1,457 (20.58)		267 (3.30)	3,257 (16.07)	
*Drug-related variables*, number (%)								
Hypertension drug treatment	2,626 (36.86)	2,600 (36.75)	2,637 (37.27)	2,447 (34.57)	0.004	3,167 (39.14)	7,143 (35.25)	<0.001
*Comorbidity*, number (%)								
Obesity (BMI ≥27)	2,688 (37.73)	2,640 (37.31)	2,635 (37.24)	2444 (34.53)	<0.001	3,022 (37.35)	7,385 (36.45)	0.16
CAD	533 (7.48)	550 (7.77)	558 (7.89)	519 (7.33)	0.58	623 (7.70)	1,537 (7.59)	0.76
CHF	138 (1.94)	146 (2.06)	155 (2.19)	150 (2.12)	0.75	169 (2.09)	420 (2.07)	0.97
Cancer	119 (1.67)	135 (1.91)	138 (1.95)	178 (2.51)	0.003	174 (2.15)	396 (1.95)	0.31
Hyperlipidemia	1,894 (26.58)	1,950 (27.56)	1,836 (25.95)	1,671 (23.61)	<0.001	2,063 (25.50)	5,288 (26.10)	0.31
Hypertension	2,969 (41.67)	3,056 (43.19)	3,165 (44.73)	2,878 (40.66)	<0.001	3,669 (45.35)	8,399 (41.45)	<0.001
Atrial fibrillation	25 (0.35)	28 (0.40)	29 (0.41)	33 (0.47)	0.75	34 (0.42)	81 (0.40)	0.89
Chronic hepatitis	706 (9.91)	706 (9.98)	736 (10.40)	733 (10.36)	0.68	832 (10.28)	2,049 (10.11)	0.68
COPD	256 (3.59)	269 (3.80)	307 (4.34)	346 (4.89)	<0.001	366 (4.52)	812 (4.01)	0.05
*Blood biochemical indices*, mean (SD)								
Triglyceride (mg/dL)	162.41 (123.25)	164.98 (123.58)	174.79 (136.19)	188.96 (158.34)	<0.001	153.12 (111.28)	180.61 (144.54)	<0.001
High-density lipoprotein (mg/dL)	46.91 (14.09)	46.46 (13.74)	46.77 (14.32)	46.58 (14.90)	0.24	46.51 (14.35)	46.75 (14.23)	0.21
Low-density lipoprotein (mg/dL)	116.93 (30.11)	117.06 (30.77)	117.17 (31.09)	117.17 (31.87)	0.96	113.73 (29.66)	118.43 (31.36)	<0.001
Fasting plasma glucose (mg/dL)	157.29 (45.93)	161.31 (44.57)	169.64 (46.15)	183.82 (53.34)	<0.001	134.26 (28.12)	181.47 (48.63)	<0.001
HbA1c (%)	7.55 (1.52)	7.78 (1.48)	8.13 (1.51)	8.51 (1.58)	<0.001	6.30 (0.51)	8.67 (1.32)	<0.001
eGFR (mL/min/1.73 m^2^)^a^	76.53 (20.70)	76.03 (21.21)	74.78 (21.93)	73.87 (23.42)	<0.001	72.59 (21.14)	76.40 (22.05)	<0.001

^a^Missing data, number = 576. Differences in continuous variables were tested by Student’s *t*-test and ANOVA. Differences in categorical variables were tested by Chi-square test. ANOVA: analysis of variance; BMI, body mass index; CAD: coronary artery disease; CHF: congestive heart failure; COPD: chronic obstructive pulmonary disease; CV: coefficient of variation; eGFR: estimated glomerular filtration rate; FPG: fasting plasma glucose; HbA_1_c, glycated hemoglobin; SD, standard deviation.

Table 2 shows HRs for ischemic stroke in subjects grouped by quartiles of FPG-CV and various HbA_1_c levels. Compared to patients with the first quartile, age-gender adjusted HRs in the fourth, third and second FPG-CV quartiles were 1.57 (95% CI 1.40, 1.77), 1.44 (1.27, 1.62) and 1.24 (1.10, 1.41), respectively. Considering lifestyles, comorbidity and complications, the FPG-CV effect was slightly attenuated but still significant. Compared to patients with an HbA_1_c level <7%, those with an HbA_1_c level 7% to 8%, 8% to 9% and ≥9% manifested greater risk (age-gender adjusted HR: 1.27 (1.13, 1.43), 1.55 (1.37, 1.75) and 2.06 (1.85, 2.31), respectively). Similarly, the effect of HbA_1_c was slightly attenuated after multivariate adjustment. We noted linear trends across both FPG-CV and HbA_1_c categories. Gender-specific associations of HbA_1_c and FPG-CV and ischemic stroke yield similar findings. Multivariate-adjusted HRs for the third and fourth FPG-CV quartiles were 1.38 (1.16, 1.64) and 1.36 (1.14, 1.62), respectively, in women; 1.08 (0.91, 1.28) and 1.19 (1.003, 1.40), respectively, in men. Multivariate-adjusted HRs for patients with HbA_1_c levels 8% to 9%, and ≥9% were 1.29 (1.07, 1.56) and 1.65 (1.37, 1.98), respectively, in women; 1.37 (1.14, 1.63) and 1.70 (1.43, 2.03), respectively, in men.

**Table 2:** **Hazard ratios (HRs) of ischemic stroke for quartiles of FPG-CV and HbA**_**1**_ **c levels (n = 28,354)**

	**Number**	**Cases**	**Person-years**	**IR**	**Ischemic stroke (Number = 2,250)**	***P*** **for trend**
**Variables**					**HR (95%CI)**	
Age and gender-adjusted (the first multivariate model)						
FPG-CV (%)						<0.001
≤14.1	7,125	489	55,218.23	8.86	1.00	
14.1 to 25.2	7,075	545	53,865.12	10.12	1.24 (1.10, 1.41)***	
25.2 to 42.0	7,076	596	52,764.96	11.30	1.44 (1.27, 1.62)***	
>42.0	7,078	620	51,198.11	12.11	1.57 (1.40, 1.77)***	
HbA_1_c (%)						<0.001
<7.0	8,091	537	61,419.14	8.74	1.00	
7.0 to 8.0	7,607	566	57,820.08	9.79	1.27 (1.13, 1.43)***	
8.0 to 9.0	5,800	490	43,589.18	11.24	1.55 (1.37, 1.75)***	
≥9.0	6,856	657	50,218.02	13.08	2.06 (1.85, 2.31)***	
Multivariate-adjusted^a^ (the second multivariate model)						
FPG-CV (%)						<0.001
≤14.1	7,125	489	55,218.23	8.86	1.00	
14.1 to 25.2	7,075	545	53,865.12	10.12	1.13 (1.00, 1.27)	
25.2 to 42.0	7,076	596	52,764.96	11.30	1.24 (1.10, 1.40)***	
>42.0	7,078	620	51,198.11	12.11	1.30 (1.15, 1.47)***	
HbA_1_c (%)						<0.001
<7.0	8,091	537	61,419.14	8.74	1.00	
7.0 to 8.0	7,607	566	57,820.08	9.79	1.14 (1.00, 1.29)*	
8.0 to 9.0	5,800	490	43,589.18	11.24	1.32 (1.16, 1.50)***	
≥9.0	6,856	657	50,218.02	13.08	1.65 (1.45, 1.87)***	
Multivariate-adjusted^b^ (the third multivariate model)						
FPG-CV (%)						<0.001
≤14.1	7,125	489	55,218.23	8.86	1.00	
14.1 to 25.2	7,075	545	53,865.12	10.12	1.11 (0.98, 1.25)	
25.2 to 42.0	7,076	596	52,764.96	11.30	1.22 (1.08, 1.38)**	
>42.0	7,078	620	51,198.11	12.11	1.27 (1.12, 1.43)***	
HbA_1_c (%)						<0.001
<7.0	8,091	537	61,419.14	8.74	1.00	
7.0 to 8.0	7,607	566	57,820.08	9.79	1.14 (1.01, 1.29)*	
8.0 to 9.0	5,800	490	43,589.18	11.24	1.33 (1.17, 1.51)***	
≥9.0	6,856	657	50,218.02	13.08	1.68 (1.48, 1.90)***	
FPG-CV (%)						0.009
≤14.1	7,125	489	55,218.23	8.86	1.00	
14.1 to 25.2	7,075	545	53,865.12	10.12	1.09 (0.96, 1.23)	
25.2 to 42.0	7,076	596	52,764.96	11.30	1.16 (1.03, 1.31)*	
>42.0	7,078	620	51,198.11	12.11	1.17 (1.03, 1.32)*	
HbA_1_c (%)						<0.001
<7.0	8,091	537	61,419.14	8.74	1.00	
7.0 to 8.0	7,607	566	57,820.08	9.79	1.13 (1.00, 1.27)	
8.0 to 9.0	5,800	490	43,589.18	11.24	1.30 (1.14, 1.47)***	
≥9.0	6,856	657	50,218.02	13.08	1.63 (1.43, 1.85)***	

*P <0.05; **P < 0.01; ***P <0.001. Multivariate-adjusted ^a^for age, gender, tobacco, alcohol, duration of diabetes, type of hypoglycemic drugs, hypertension drug treatment and obesity.

Multivariate-adjusted ^b^for coronary artery disease, congestive heart failure, cancer, hyperlipidemia, hypertension, atrial fibrillation, chronic hepatitis, chronic obstructive pulmonary disease and estimated glomerular filtration rate (eGFR) in addition to the variables in the second multivariate model. FPG-CV: coefficient of variation of fasting plasma glucose; HbA_1_c, glycated hemoglobin; IR: incidence density rate = number of incident cases/person-years*1,000.

**Figure 3:**
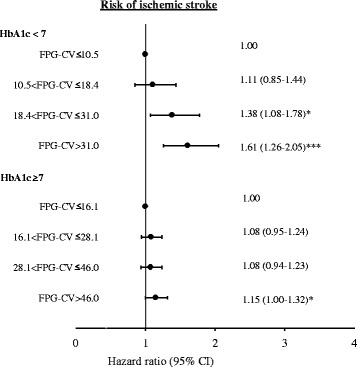
**Risks of ischemic stroke for FPG-CV stratified by HbA**_**1**_ **c (<7.0 or ≥7.0) in type 2 diabetic patients enrolled in the National Diabetes Care Management Program, Taiwan.** *: *P* <0.05; ***: *P* <0.001. FPG-CV: coefficient of variation of fasting plasma glucose; HbA1c, glycated hemoglobin.

With FPG-CV and HbA_1_c considered simultaneously, both manifested a significant effect on ischemic stroke: multivariate-adjusted HRs for the fourth and third FPG-CV quartiles were 1.16 (1.03, 1.31) and 1.17 (1.03, 1.32), respectively; for HbA_1_c levels 8% to 9%, and ≥9% were 1.30 (1.14, 1.47) and 1.63 (1.43, 1.85), respectively. Sensitivity analysis assessed potential bias due to comorbidity, excluding patients with hyperglycemic hyperosmolar nonketotic coma (n = 355), diabetic ketoacidosis (n = 216), myocardial infarction (n = 653), atrial fibrillation (n = 115), hypoglycemia (n = 80) and all comorbidity (n = 1,346) (Table 3). A similar significant association was found for the third and fourth FPG-CV quartiles and for HbA_1_c levels 7.0% to 8.0%, 8.0% to 9.0%, and ≥9.0%. To rule out the impact of insulin by excluding patients who use it, multivariate-adjusted HRs for the third and fourth FPG-CV quartiles were 1.22 (1.08, 1.38) and 1.29 (1.14, 1.46), respectively. With FPG-CV subgrouped based on deciles, multivariate-adjusted HRs for FPG-CV levels 20.4% to 25.2%, 25.2% to 30.5%, 30.5% to 37.6%, 37.6% to 47.3%, 47.3% to 65.3%, and >65.3% were 1.36 (1.13, 1.65), 1.35 (1.12, 1.64), 1.41 (1.17, 1.71), 1.39 (1.15, 1.68), 1.38 (1.14, 1.67) and 1.41 (1.16, 1.71), respectively. To rate the impact of potential false positives for diabetes on the findings, further analysis excluded those who were not on medication. Multivariate-adjusted HRs for the third and fourth FPG-CV quartiles were similar (1.26 (1.12, 1.43) and 1.32 (1.17, 1.50), respectively). To rule out the confounding effect of HbA_1_c, FPG-CV stratified by HbA_1_c (<7.0% or ≥7%) was considered (Figure 3). We found no significant interaction effects of FPG-CV and HbA_1_c. Multivariate-adjusted HRs for the third and fourth FPG-CV quartiles showed significant linkage with ischemic stroke in patients with HbA_1_c <7.0%; the FPG-CV fourth quartile was significantly correlated with ischemic stroke in patients with HbA_1_c ≥7.0% (1.15 (1.00, 1.32)).

**Table 3:** **Sensitivity analysis for evaluating bias due to comorbidity**

		**Ischemic stroke HR (95%CI)**			
		**FPG-CV (%)**			
**Model** ^**a**^	**Number**	**≤14.1**	**14.1 to 25.2**	**25.2 to 42.0**	**>42.0**
Model I	27,999	1.00	1.10 (0.97, 1.24)	1.21 (1.08, 1.37)**	1.27 (1.12, .144)***
Model II	28,138	1.00	1.12 (0.99, 1.26)	1.22 (1.09, 1.38)**	1.27 (1.13, 1.44)***
Model III	27,701	1.00	1.12 (0.99, 1.27)	1.22 (1.08, 1.38)**	1.27 (1.12, 1.44)***
Model IV	28,239	1.00	1.11 (0.98, 1.26)	1.22 (1.08, 1.37)**	1.28 (1.13, 1.44)***
Model V	28,274	1.00	1.11 (0.98, 1.26)	1.22 (1.08, 1.38)**	1.27 (1.13, 1.44)***
Model VI	27,008	1.00	1.12 (0.99, 1.27)	1.22 (1.08, 1.38)**	1.29 (1.14, 1.46)***
		**HbA1c (%)**			
**Model** ^**a**^	**Number**	**<7.0**	**7.0 to 8.0**	**8.0 to 9.0**	**≥9.0**
Model I	27,999	1.00	1.15 (1.02, 1.30)*	1.34 (1.18, 1.53)***	1.67 (1.47, 1.90)***
Model II	28,138	1.00	1.14 (1.01, 1.28)*	1.33 (1.17, 1.51)***	1.67 (1.46, 1.89)***
Model III	27,701	1.00	1.13 (1.00, 1.28)*	1.33 (1.16, 1.51)***	1.68 (1.48, 1.91)***
Model IV	28,239	1.00	1.13 (1.00, 1.28)*	1.34 (1.17, 1.52)***	1.68 (1.48, 1.91)***
Model V	28,274	1.00	1.15 (1.02, 1.30)*	1.34 (1.18, 1.52)***	1.70 (1.49, 1.92)***
Model VI	27,008	1.00	1.14 (1.01, 1.29)*	1.36 (1.19, 1.55)***	1.69 (1.48, 1.93)***

*P <0.05; **P <0.01; ***P <0.001. Multivariate-adjusted ^a^for age, gender, tobacco, alcohol, duration of diabetes, hypoglycemic drugs, hypertension drug treatment, obesity, coronary artery disease, congestive heart failure, cancer, hyperlipidemia, hypertension, atrial fibrillation, chronic hepatitis, chronic obstructive pulmonary disease and estimated glomerular filtration rate (eGFR). Model I excluding patients with hyperglycemic hyperosmolar nonketotic coma (HHNK) (Number = 355). Model II excluding patients with diabetic ketoacidosis (DKA) (Number = 216). Model III excluding patients with myocardial infarction (Number = 653). Model IV excluding patients with atrial fibrillation (Number = 115). CI: confidence interval; FPG-CV: coefficient of variation of fasting plasma glucose; HR: hazard ratio; HbA1c, glycated hemoglobin.

## Discussion

This study is the first to demonstrate variation in FPG measurement predicting ischemic stroke in type 2 diabetic patients more than 30 years old. Strengths of this study include a relatively large number of type 2 diabetic cases, standard data collection procedures, a sufficiently long follow-up period and available information on large numbers of potential confounding factors. Our results show that FPG-CV is a glucose variation measure that pinpoints the association between oscillating plasma glucose and ischemic stroke independent of HbA_1_c. Our findings are relevant to the clinical management of type 2 diabetes. FPG should be measured to monitor glycemic variability as well as HbA_1_c. Therapies now used should be evaluated for their potential to minimize glucose fluctuation and/or reduce HbA_1_c in type 2 diabetic patients to prevent ischemic strokes.

HbA_1_c level reflects average glucose over the preceding eight to twelve weeks of glycemic control and is viewed as a more accurate and stable measure than fasting blood glucose level [21,22]. Evidence points to elevated HbA_1_c as an independent risk factor for ischemic stroke [23,24]. However, meta-analyses and recent randomized controlled trials such as the Action to Control Cardiovascular Risk in Diabetes (ACCORD) trial, the Action in Diabetes and Vascular Disease (ADVANCE) trial, and the Veterans’ Administration Diabetes Trial (VADT) found that lowering blood glucose did not appreciably reduce the pooled incidence of stroke [5,25-27]. Interpretation is complicated, partly because HbA_1_c is just one aspect of glycemic disorder: as an integrated measure of sustained chronic hyperglycemia, it fails to reflect glucose variability and the risks associated with extreme glucose swings [28]. Patients with similar HbA_1_c levels show markedly variant daily glucose excursions. Increasing evidence hints that glucose variability raises the risk of diabetic complications [8,29-31]. Consistent with previous studies, we found that HbA_1_c is independently associated with ischemic stroke [19,21]. We saw a novel predictor representing glucose instability (FPG-CV) portending greater risk of ischemic stroke. We hypothesize that the failure of therapy targeting chronic sustained hyperglycemia on ischemic stroke may be due to the fact that controlling fasting glucose and HbA_1_c but not glycemic variability may be inadequate. Lessened glycemic variability might improve outcome; stability of fasting plasma glucose over time should be a goal in preventing ischemic stroke. A well-designed study that entails stablizing the glucose level will verify whether glucose stability reduces ischemic stroke.

Both the Verona Diabetes and our own studies prove that FPG-CV is an independent predictor of total, cancer and cardiovascular mortality [11-15]. A strong correlation between glucose variability and mortality in critically ill patients emerged from a systemic review with at least 12 independent cohorts [32]. Stroke, like macroangiopathy, is a facet of cardiovascular disease in diabetes, poorly recognized as a specific target for evaluation.

We noted links between FPG variation and ischemic stroke, with three possible explanations. First, plausible mechanisms explain linkage of HbA_1_c and FPG variation with ischemic stroke. Oscillating plasma glucose proves more deleterious than constant high glucose on endothelial function and oxidative-stress generation, factors in macroangiopathy and, thus, predisposition to ischemic stroke [33]. Glucose fluctuations appear more relevant to atherosclerosis progression in type 2 diabetics than those with sustained hyperglycemia [34,35]. Recent studies indicate that change in intima-media thickness, a surrogate marker for early cerebral atherosclerosis, is associated with a reduction in daily glucose excursions, but not indices of chronic sustained hyperglycemia [36].

Second, oscillating plasma glucose can predispose to hypoglycemia, which may act as a precipitating factor of cerebrovascular events [37,38]. It has also been shown that hyperglycemia after hypoglycemia could be more dangerous than that when hypoglycemia is followed by normoglycemia [39]. In this regard, patients with well-documented hypoglycemic episodes were more represented among subjects in the fourth quartile of FPG-CV. Yet after sensitivity analysis by excluding patients with hypoglycemia, FPG-CV showed links with incidence of ischemic stroke; this association cannot be explained by hypoglycemia in patients with high glucose variability. One could speculate FPG-CV and HbA_1_c as strongly interrelated, that FPG-CV seems an epiphenomenon of poor glycemic control. With no significant interactions of FPG-CV and HbA_1_c on ischemic stroke observed, it seems that FPG-CV and HbA_1_c describe separate aspects of dysglycemic impact on ischemic stroke.

Third, it is possible that glucose variation reflects the effects of coexisting conditions and incompletely quantified confounding variables that can heighten risk of ischemic stroke rather than directly cause it [13-15]. Diabetic patients, vulnerable to sugar variability, showed more comorbidity: for example, hypertension, hyperlipidemia, obesity. Baseline coexisting illness, comorbidity and complications were considered in our regression models; patients who had glucose instability with these co-factors were excluded in sensitivity analysis to disprove this possibility. Strong correlation of FPG-CV with ischemic stroke remained the same, independent of other risk factors.

This study has limitations. First, findings were limited by potential residual and unrecognized confounding variables, since this study was observational. Second, measurement errors were possible due to the large amount of data gathered from clinical practice. Third, information on subtype or size of infarction was not available. The effect of glucose variability in ischemia subtypes must be examined in future studies. Fourth, we only have one-year FPC measurements in the NDCMP dataset to estimate FPC-CV. We thus could not evaluate the effect of FPC-CV during follow-up on risk of ischemic stroke. Fifth, assessment of glucose variability is complex; quantification of glucose variability by prior studies, including FPG-CV, shows limitations [6,7,15,40]. No ‘gold standard’ currently exists to rate glucose variability. One point-counterpoint article regarding glucose variability cited CV and mean absolute glucose change as better markers for glucose variability [41]. Indeed, assays of fasting plasma glucose with CV to gauge cardiovascular risk in clinical practice are more feasible than those more complex methods, so significant to validate their utility. Future study must compare predictive capacity of glucose variance for medical outcome in diabetic cases. Finally, a growing body of evidence shows a causal relation between post-prandial hyperglycemia and cardiovascular disease independent of HbA_1_c and FPG [42,43]. This study did not measure post-prandial glucose and, thus, could not assess the effect of post-prandial hyperglycemia contributing to ischemic stroke. We elucidated how, in addition to HbA_1_c, FPG-CV variation predicts ischemic stroke in type 2 diabetics. Special heed should be paid to maintain glucose concentration. Clinical trials with large sample sizes entailing intervention to stabilize glucose level should unearth evidence that glucose stability lowers the incidence of ischemic stroke.

## Conclusions

Besides HbA_1_c, FPG-CV was a potent predictor of ischemic stroke in type 2 diabetic patients, suggesting that different therapeutic strategies now in use be rated for their potential to (1) minimize glucose fluctuations and (2) reduce HbA_1_c level in type 2 diabetic patients to prevent ischemic stroke.
